# Taste Sensitivity Is Associated with Food Consumption Behavior but not with Recalled Pleasantness

**DOI:** 10.3390/foods8100444

**Published:** 2019-09-27

**Authors:** Sari Puputti, Ulla Hoppu, Mari Sandell

**Affiliations:** 1 Functional Foods Forum, University of Turku, Turku 20014, Finland; sari.puputti@utu.fi (S.P.); ulla.hoppu@utu.fi (U.H.); 2 Monell Chemical Senses Center, 3500 Market Street, Philadelphia, PA 19104, USA

**Keywords:** taste sensitivity, behavior, food, perception, consumption, pleasantness

## Abstract

As taste perception varies between individuals, it might be important in explaining food consumption behavior. Previous studies have focused on sensitivity to the bitter tastant PROP (6-n-propylthiouracil) concerning eating with little attention paid to other tastants. For the first time, connections between food consumption behavior, pleasantness, and taste sensitivity are studied with five taste modalities. Sensitivity to bitterness, sourness, umami, saltiness, and sweetness as well as an overall taste sensitivity score was determined with intensity evaluation for 199 Finnish adults. Recalled pleasantness and food consumption behavior were enquired with online questionnaires. Consumption concerned intake of vegetables, fruits, and berries; use-frequency of specific foods; and tendency to mask or modify tastes of foods. All modality-specific taste sensitivities were related to some consumption behavior but none to recalled pleasantness. A higher taste sensitivity score indicated avoidance of coffee, lower consumption of pungent foods, and a more frequent habit of adding ketchup to a meal. In conclusion, it may be more informative to study the influence of taste sensitivity on food consumption behavior with taste modalities separately rather than with a general indicator of taste sensitivity. Additionally, these results highlight the importance of studying actual behavior toward food and not just liking.

## 1. Introduction

The taste of food is a key factor in food choice. As taste perception varies between individuals, individual taste perception might be important regarding food choice, personal nutrition, and, further, quality of life and development of chronic diseases. Thus, wellbeing may be improved and chronic diseases controlled by considering the taste of foods that are consumed regularly. Then again, the consumption of vegetables, fruits, and berries (VFB) is essential for health and wellbeing. Nevertheless, VFB are consumed less than recommended; in Finland, only every tenth man and every fifth woman ate VFB the recommended amount (500 g per day) in 2017, and the consumption has decreased during recent years [1]. This unfavorable behavior may be partly due to the taste of VFB that does not attract all people. At the same time, consuming more calories than expending, especially consuming energy-dense foods and beverages, is increasing the prevalence of overweight individuals and obesity as well as associated morbidity [2]. To promote a healthier diet, individual motives behind food choices must be investigated.

People can perceive at least five taste modalities: sour, bitter, sweet, salty, and umami. These tastes are perceived individually, and most variation seems to occur in umami and bitter perception [3,4,5]. Individual taste sensitivity can be measured using several psychophysical methods [6]. Threshold sensitivity measures the lowest concentration of a tastant that is either detected (detection threshold) or identified correctly (recognition threshold). The threshold concentrations are typically very low and thus, may be irrelevant in explaining food liking or consumption [7,8]. Another commonly used measurement of taste perception is intensity rating. The subjects evaluate the intensity of sensation elicited by a tastant at a certain concentration level. The concentration is typically above the threshold. The third commonly used measure is a PROP (6-n-propylthiouracil) taster status. It is a phenotype related to a bitter receptor genotype *TAS2R38* at least. Sensitivity to PROP has been applied to classify subjects as supertasters, medium tasters, and non-tasters [9]. In addition, PROP bitterness intensity has been found to correlate with intensity perception of other tastes [10,11,12,13], suggesting that the PROP taster status might represent general taste sensitivity, although some have challenged this view [5,6,11,14]. Considering the taste perception of food, intensity measures of PROP or other tastants may be more relevant measures of individual taste sensitivity than the threshold measures [8].

Little is known about the association between taste sensitivity and food pleasantness or food consumption behavior, such as consumption frequency, or habits to mask tastes in food (for example, masking the bitter taste of coffee with milk or sugar). Higher sweet sensitivity indicated a lower intake of sweet foods and a lower liking of some sweet beverages [7]. However, in other studies, sweet sensitivity was not related to sweet food-related behavior [15,16], such as to the importance of adding sugar in coffee or tea [16]. Lipchock et al. (2017) [17] reported that daily coffee consumers were more sensitive to caffeine, the bitter compound found in coffee than those who consumed coffee rarely or not at all. Furthermore, salty and sour taste perception correlated with alcohol intake, but they were not significant predictors of alcohol intake in a multivariate-adjusted model [18]. Research has focused on PROP taste [19,20,21,22,23] and taste genetics [21,24,25,26,27] concerning food consumption behavior and liking. Thus, it is essential to investigate using other tastants whether taste sensitivity is related to food pleasantness and consumption.

This study is part of a large research project concerning individual differences in sensory perception and food-related behavior. Previously, we reported inter-individual variations in color [28] and taste perception [5,29]. The study population was segmented into taste sensitivity groups for each taste modality (sour, bitter, sweet, salty, and umami) based on intensity judgments of aqueous solutions [5]. The objective of this study was to investigate further whether taste sensitivity is associated with food consumption behavior and recalled pleasantness of certain foods and beverages typical to the Finnish food culture. The studied consumption habits included weekly intake of VFB (as a number of portions), habits regarding the masking or modifying the taste of food, and use-frequency of specific foods and beverages. In addition to taste sensitivity, sex, age, education, and BMI were studied as possible explanatory factors for food consumption and pleasantness.

## 2. Materials and Methods

### 2.1. Participants

The participants were recruited by announcements at the University of Turku and at public events. In total, 206 Finnish-speaking volunteers (19–79 years) participated in the study. The exclusion criteria included pregnancy and being in a lactating state. Additionally, one person was excluded afterward because of self-reported ageusia after head trauma. Smoking was not an exclusion criterion, but as only six subjects reported themselves to be daily smokers, they were excluded from this study. Otherwise, all volunteers (*N* = 199) were selected for the study without prerequisites for a balanced sample regarding any subject characteristic. After a full account of research aims, written informed consent was provided by all of the subjects. They were rewarded with food products after every visit. The study was approved by the Southwest Finland Hospital District’s Ethics Committee (145/1801/2014), and it has been performed in accordance with the 1964 Declaration of Helsinki and its later amendments.

### 2.2. Taste Sensitivity

The measuring of taste sensitivity was reported in detail earlier in Puputti et al. [5]. In summary, taste sensitivity was determined using four concentration levels of prototypical elicitors of sour, bitter, sweet, salty, and umami sensations (Table 1). The intensity judgments were evaluated on line scale (range from 0 = not at all to 10 = extremely strong) and were analyzed with hierarchical clustering. Three sensitivity groups were formed for each taste modality. Thus, for each taste, there was a group of the least sensitive (cluster 1), the semi-sensitive (cluster 2), and the most sensitive tasters (cluster 3). In addition to the taste modality-specific sensitivity, general taste sensitivity was analyzed using a taste sensitivity score. The score was determined as the mean of the taste modality-specific cluster memberships (score range 1.0–3.0). The closer the score was to 3, the more sensitive the participant.

The sensory test was executed in the sensory evaluation laboratory (ISO8589) of Functional Foods Forum, University of Turku. The subjects were instructed to refrain from food, beverages other than water, chewing gum, and smoking for at least 1 h prior to testing. The responses were collected with Compusense *five* plus software (Compusense Inc., Guelph, ON, Canada).

### 2.3. Questionnaires

Webropol online questionnaires (Webropol Inc, Helsinki, Finland) were used for the data collection of subject characteristics, food consumption behavior, and recalled pleasantness of foods and beverages. Sex was changed to a dummy variable: 0 = male, 1 = female. Age was not normally distributed, so it was divided into three categories: the youngest, 19–34 years old (M (SD) = 27.8 (4.1) years); the middle-aged, 35–49 years old (M (SD) = 42.5 (4.3) years); and the oldest, 50–79 years old (M (SD) = 61.9 (8.6) years). BMI was calculated from self-reported height and weight according to the formula kg/(m)^2^. BMI also had a non-normal distribution and the participants were divided into three categories: the lean subjects (BMI < 25.0, M (SD) = 21.8 (2.0)) including three underweight persons (BMI < 18.5), the overweight subjects (BMI = 25.0–29.9, M (SD) = 27.2 (1.5)), and the obese subjects (BMI ≥ 30.0, M (SD) = 34.7 (4.2)).

#### 2.3.1. Portions of VFB per Week

The frequency of consumption of vegetables, fruits, and berries was inquired about separately for each food category, with the response options being “every day,” “5–6 days per week,” “3–4 days per week,” “1–2 days per week,” and “more seldom than once per week”. Additionally, the typical number of portions of vegetables, fruits, and berries consumed per day was inquired about using a category scale (0–6 portions), with an additional response option of “I cannot say.” One portion was described as one carrot, tomato, or apple, or 100 mL of berries or grated vegetables. From the answers to these questions, a new variable called portions per week (range 0–42) was computed separately for vegetables, fruits, and berries as portions per day multiplied by use-frequency (mean).

#### 2.3.2. Masking and Modifying Taste

The frequency of certain consumption habits was thought to describe the tendency to mask or modify taste of food. The questions started with “How frequently do you…?” and dealt with masking bitterness: (1) add milk to coffee, (2) add cream to coffee, (3) add sugar to coffee, (4) add sweetener to coffee, (5) add sugar or honey to tea, (6) add sweetener to tea, (7) add milk to tea; modifying taste with salt or condiments: (8) add salt to water when cooking vegetables, (9) add salt to a meal when eating it, (10) add aromatic salt (mixture of salt and seasoning) to a meal when eating it, (11) add ketchup to a meal when eating it, (12) add soy sauce to a meal when eating it; and masking bitterness, sourness and astringency of berries: (13) add sugar, honey or something else sweet to berries. The response options were “always,” “often,” “sometimes,” “rarely,” “never,” and when appropriate “I don’t drink coffee/drink tea/prepare food.” The responses “I don’t drink coffee/drink tea/prepare food” were removed (marked as missing) before statistical analysis but two new dichotomous variables were also formed: drink coffee vs. do not drink coffee, and drink tea vs. do not drink tea.

#### 2.3.3. Recalled Pleasantness and Use-Frequency of Foods and Beverages

Recalled pleasantness and use-frequency of specific foods and beverages (*N* = 58) belonging to the Finnish food culture and eliciting diverse sensory experiences were inquired about to investigate liking and consumption habits. The selection of certain items was also based on the assumption that their sensory profiles divide consumers’ opinions strongly. Pleasantness ratings were investigated with a 9-point hedonic scale (1 = extremely unpleasant, 9 = extremely pleasant). The response option “I cannot say” was included in the case of an unfamiliar food or beverage. These responses were removed (marked as missing) before statistical analyses. Consumption frequencies of the same food and beverage items were inquired about using a 6-point category scale with the response options “daily,” “a few times per week,” “once per week,” “once or twice per month,” “a few times per year,” and “more seldom or never.”

### 2.4. Statistics

A chi-squared test or Fisher’s Exact Test (FET) was applied to analyze the associations between the categorical variables. A t-test or ANOVA (Tukey as a *post hoc* test or Tamhane’s test if variances were not equal) was applied to compare differences between groups. If the assumptions for the parametric methods were not met, the Kruskal-Wallis and/or the Mann-Whitney U test was applied. Bonferroni correction was applied for multiple comparisons when appropriate. Associations between the taste sensitivity score and the variables concerning the habits of masking/modifying taste and the weekly portions of fruits and berries were analyzed with the Spearman rank correlation whereas the association between the taste sensitivity score and the weekly portions of vegetables was analyzed with the Pearson correlation.

Recalled pleasantness ratings for food and beverage categories were subjected to factor analysis. The categories comprised vegetables (bitter, pungent, mild), vegetable dishes, and pungent condiments (*N* = 20); fruits and berries (*N* = 13); sweet, salty, and fatty foods (*N* = 13); and alcoholic and non-alcoholic beverages (*N* = 12) (original items are listed in Supplementary Material Table S1). The principal component method was applied for component extraction and varimax rotation to gain more interpretable results. The number of factors was decided based on an Eigenvalue greater than 1, the scree plots inspection, and meaningful component content. Variables possessing communality (estimate of variance in a variable accounted for by the extracted components) under 0.300 were removed from the model to create a better model. Component scores for further analyses were obtained by the regression method.

The associations between the background factors, taste sensitivity, and the pleasantness components from factor analysis were analyzed using the hierarchical multivariate linear regression. In the first block, sex and age were entered into the model. In the second model, BMI group and/or education were added to the model if they possessed a significant contribution to the model after controlling for sex and age. In the third block, sour, bitter, sweet, salty and/or umami sensitivity in one model or the taste sensitivity score in another model were entered into the final model if they possessed a significant contribution after controlling for the previously entered predictors. For the second and third block, the forward method was applied to obtain the simplest model. The criterion for including a variable was the significance of the regression coefficient at a *p* ≤ 0.1 level. This hierarchical approach enabled the investigation of whether BMI and education in the second block enhanced the prediction model and whether taste sensitivities in the third block enhanced the previous model.

Following the categories of the pleasantness components, new use-frequency variables were calculated as the mean of the consumption frequency of the pleasantness component items. Thus, the pleasantness components and the new use-frequency components comprised the same food or beverage items. The correlations between pleasantness and use-frequency were analyzed using the Pearson correlation. The hierarchical multivariate linear regression approach was also applied to the new use-frequency components to investigate whether factors other than the pleasantness score explained consumption. The process was similar to the pleasantness component analysis except that the third block consisted of the equivalent pleasantness component because it was expected to have a major contribution to the model. Consequently, taste sensitivities were added in the fourth block. The forward method was applied for blocks 2–4.

The criterion for significance was set to be *p* < 0.05. All statistical analyses were completed with IBM SPSS Statistics 25.0 (IBM Corporation, Armonk, NY, USA).

Some of the subjects did not complete every section of the study because of time constraints, technical issues, or self-reported hypersensitivity to caffeine. Missing data were dealt with in each analysis rather than entirely excluding the subjects with missing data. The subject numbers included in the analyses are provided in the text, tables, and figures.

## 3. Results

### 3.1. Subject Characteristics

The subjects’ (*N* = 199) characteristics are presented in Table 2. Age and BMI were related (*Χ*^2^ (4) = 25.3, *p* < 0.001) as the majority (77.1%) of the youngest individuals were lean whereas under half of the middle-aged (44.4%) or the oldest individuals (40.0%) were lean. The latter two were more likely to be overweight (33.3% of the middle-aged and 40.0% of the oldest individuals) than the youngest individuals (10.8%). Otherwise, sex, age, BMI, or education were not related. The mean taste sensitivity score was 1.94 (SD 0.50). The connections between taste sensitivities and background factors are reported in our previous publication [29]. In summary, increased age indicated lower taste sensitivity except for sweet taste. Male sex was related to lower sensitivity to sour taste and higher BMI to lower sensitivity to umami. Additionally, males had a lower taste sensitivity score than females, and the oldest subjects had a lower score than the younger subjects.

### 3.2. Portions of VFB per Week

**Figure 1:**
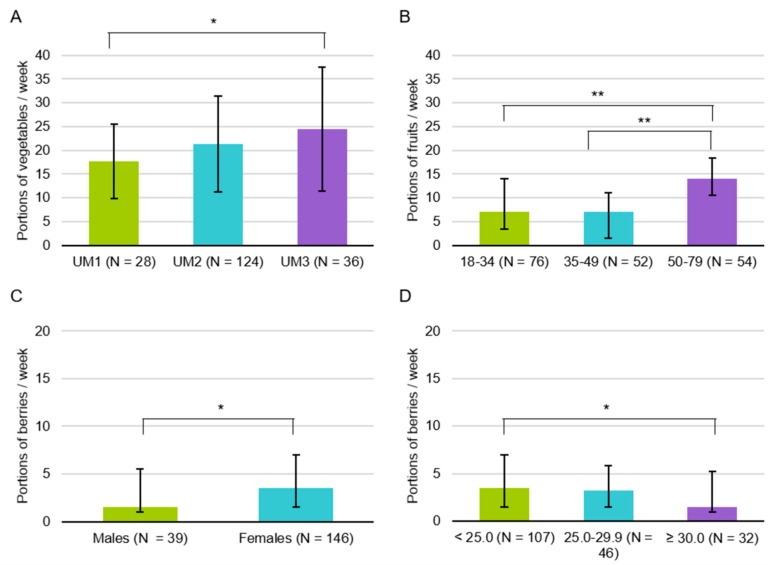
The significant group differences in the number of portions of vegetables (mean and standard deviation), fruits, and berries (median and interquartile range) per week (possible range 0-42). (**A**) vegetable portions by umami sensitivity groups, UM1 = the least sensitive, UM2 = the semi-sensitive, UM3 = the most sensitive, (**B**) fruit portions by age groups (years), (**C**) berry portions by sex, (**D**) berry portions by BMI groups. * *p* < 0.05, ** *p* < 0.001 based on the Tukey (**A**) and Mann-Whitney U (**B**–**D**) test.

The mean number of portions of vegetables consumed per week was 21.1 (SD 10.5, *N* = 177). The median number of portions of fruits per week was 10.9 (interquartile range IQR 3.5–14.0, *N* = 177) and of berries 3.5 (IQR 1.5–7.0, *N* = 177). The number of vegetable portions consumed varied depending on umami taste sensitivity (F (2) = 3.25, *p* = 0.041) (Figure 1A). Older age was related to the increased consumption of fruits (H (2) = 23.92, *p* < 0.001) (Figure 1B). Consumption of berries varied between sexes and BMI groups (U = 2246.5, *p* = 0.042, and H (2) = 7.00, *p* = 0.030, respectively) (Figure 1C,D). Education or other taste sensitivity variables were not related to the portions of VFB consumed per week.

### 3.3. Masking and Modifying Taste

The distribution of responses for some of the consumption habits was very narrow in this study population; thus, they were not analyzed further. These variables included adding cream to coffee, adding sugar to coffee, adding sweetener to coffee, adding sweetener to tea, adding milk to tea, and adding aromatic salt to a meal. The distributions for other habits are presented in Table 3. The associations were also studied separately in every age group.

**Figure 2:**
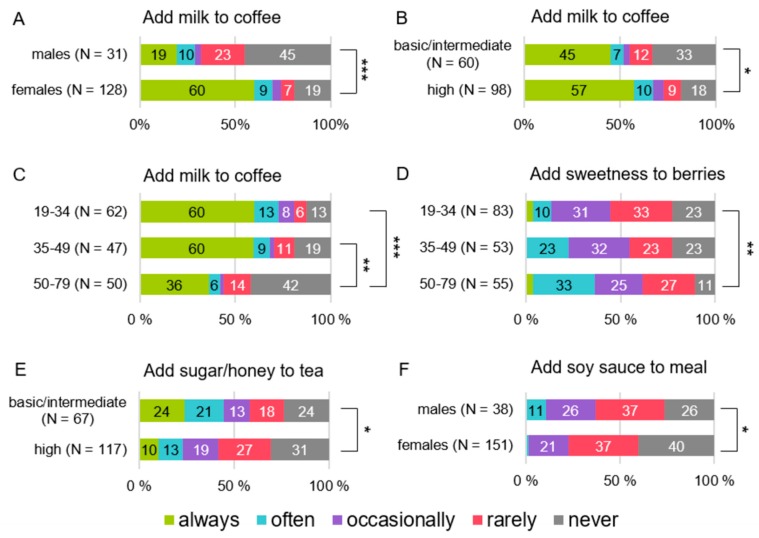
Significant group differences in frequency to mask/modify tastes. (**A**) sex vs. the habit of adding milk to coffee, (**B**) education vs. the habit of adding milk to coffee, (**C**) age (years) vs. the habit of adding milk to coffee, (**D**) age (years) vs. the habit of adding something sweet to berries, (**E**) education vs. the habit of adding sugar/honey to tea, (**F**) sex vs. the habit of adding soy sauce to a meal when eating it. * *p* < 0.05, ** *p* < 0.01, *** *p* < 0.001 based on the Mann-Whitney U test.

**Figure 3:**
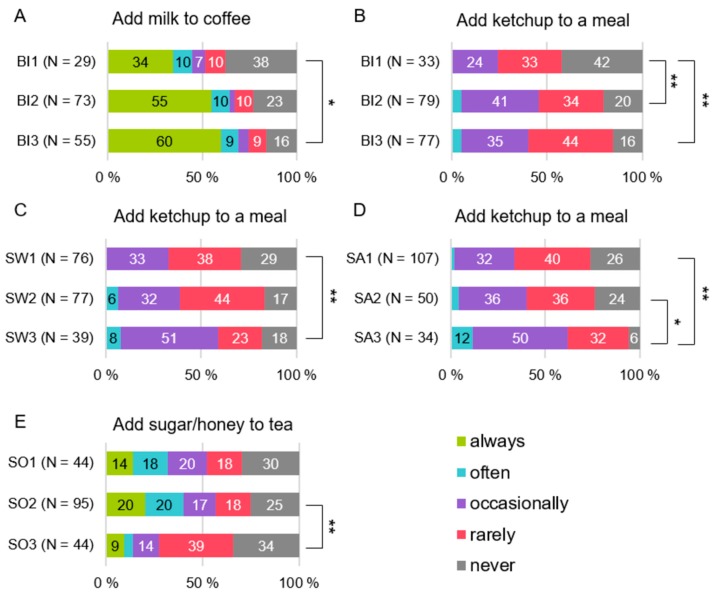
Differences in the frequency to mask/modify tastes by taste sensitivity groups, 1 = the least sensitive subjects, 2 = semi-sensitive subjects, 3 = the most sensitive subjects. (**A**) bitter sensitivity vs. the habit of adding milk to coffee, (**B**) bitter sensitivity vs. adding ketchup to a meal when eating it, (**C**) sweet sensitivity vs. adding ketchup to a meal when eating it, (**D**) salty sensitivity vs. adding ketchup to a meal when eating it, (**E**) sour sensitivity vs. habit of adding sugar/honey to tea. BI—bitter; SW—sweet; SA—salty; SO—sour. * *p* < 0.05, ** *p* < 0.01 based on Mann-Whitney U tests.

The more common habit of adding milk to coffee was related to female sex, younger age, higher education (U = 1075.5, *p* < 0.001, H (2) = 12.7, *p* = 0.002, and U = 2434.5, *p* = 0.048, respectively) (Figure 2A–C), and higher bitter sensitivity (H (2) = 6.08, *p* = 0.048) (Figure 3A).

The oldest subjects added sugar to berries more frequently than the youngest participants despite their taste sensitivity (H (2) = 7.83, *p* = 0.020) (Figure 2D).

Bitter, sweet, and salty sensitivity were related to adding ketchup to a meal when eating it (H (2) = 8.55, *p* = 0.014, H (2) = 7.56, *p* = 0.023, and H (2) = 11.8, *p* = 0.003, respectively) (Figure 3B–D). For sweet sensitivity, this was shown especially among 35–49-year-old subjects (H (2) = 8.56, *p* = 0.041) when the most sensitive to sweet used ketchup more frequently than the least sensitive (U = 44.5, *p* = 0.036). Additionally, the taste sensitivity score and adding ketchup to a meal had a statistically significant correlation (*r* = 0.178, *p* = 0.015).

Rather than the most sensitive subjects, those semi-sensitive to sourness (H (2) = 7.62, *p* = 0.022) (Figure 3E) and the lower educated subjects (U = 3754.0, *p* = 0.013) (Figure 2E) added sugar or honey to tea more frequently. Among the youngest subjects, the least sensitive to umami added sugar or honey to tea more frequently than the semi or most sensitive subjects (H (2) = 11.9, *p* = 0.008; U = 18.0, *p* = 0.028, U = 2.0, *p* = 0.013, respectively).

Males added soy sauce to a meal when eating it more frequently than females (U = 2257.0, *p* = 0.031) (Figure 2F). Among the oldest subjects, sour sensitivity was related to the habit of adding soy sauce to a meal (H (2) = 12.1, *p* = 0.007); the most sensitive to sourness added soy sauce less frequently than the least sensitive (U = 29.0, *p* = 0.027).

None of the potential predictors explained the frequency of adding salt to a meal when eating it or adding salt to vegetable cooking water.

Because a subject could also respond that he/she does not drink coffee or tea, the associations between consuming coffee or tea and taste sensitivities were analyzed. Those who avoided coffee (N = 33, 17.2% of all respondents) were more likely bitter sensitive subjects (*Χ*^2^ (2) = 12.9, *p* = 0.002) or had a higher taste sensitivity score (t (185) = 2.63, *p* = 0.009) than coffee drinkers.

### 3.4. Factor Analysis of Recalled Pleasantness

Table 4 presents the components extracted from the principal component analysis applied for the food and beverage pleasantness ratings. For further analysis, 11 composite pleasantness variables were extracted and labeled as bitter vegetables, strong-tasting vegetables, pungent foods, berries, fruits, salty-and-fatty foods, sweet-and-fatty foods, salty-and-savory foods, bitter-and-astringent alcoholic beverages, bitter-and-astringent non-alcoholic beverages, and sweet beverages.

### 3.5. Explaining Recalled Pleasantness

The associations between the pleasantness variables from factor analysis and subject characteristics were analyzed with multivariate linear regression in three steps. First, sex and age were entered. Second, BMI and education level were entered if they made a significant contribution to the model. Lastly, taste sensitivities were entered if they contributed significantly after controlling for the previously-added variables. The final models are presented in Table 5. Overall, the models explained only a relatively small proportion of the pleasantness scores: from 3.4% for sweet-and-fatty foods to 11.4% for sweet beverages. None of the taste sensitivity factors was a significant contributor to the models.

Increased pleasantness of bitter vegetables was related to male sex and lower BMI. Their contribution to the model was approximately equal (standardized β coefficients −0.172 and −0.166, respectively). Sour sensitivity was included in the model in block 3 as its *p*-value (0.067) was under the selected criterion (0.100). However, adding sour sensitivity did not enhance the model significantly when compared to the model including only sex and BMI (*R*^2^ change 0.022, F_change_ = 3.42, *p*_change_ = 0.067). Thus, the model comprising sex and BMI is reported in Table 5.

Female sex predicted a higher liking score for pungent foods. In the third step, sweet and salty sensitivities were added into the model (*p*-values 0.060 and 0.071, respectively) but their inclusion did not enhance the model comprising sex and age (for sweet *R*^2^ change 0.023, F_change_ = 3.60, *p*_change_ = 0.060; for salty *R*^2^ change 0.021, F_change_ = 3.30, *p*_change_ = 0.071).

Liking of strong-tasting vegetables, as well as berries, was explained by age as older age increased the pleasantness scores. A lower BMI was the only significant predictor of increased fruit liking.

Younger subjects had higher liking scores for salty-and-fatty foods, whereas older age predicted higher salty-and-savory foods liking. The model for sweet-and-fatty foods liking was just above the significance level, although females liked sweets more than males did. BMI was entered in the prediction models of salty-and-fatty foods and sweet-and-fatty foods but the inclusion did not enhance the models significantly (*R*^2^ change 0.019, F_change_ = 3.41, *p*_change_ = 0.067 and *R*^2^ change 0.020, F_change_ = 3.56, *p*_change_ = 0.061, respectively).

Male sex predicted higher liking scores for bitter-and-astringent alcoholic beverages and older age for bitter non-alcoholic beverages. Sweet beverage pleasantness was explained by age and BMI as younger age and higher BMI increased the pleasantness score. Based on the standardized β coefficients, age had a higher contribution to the model than BMI (−0.311 and 0.224).

### 3.6. Use-Frequency

The descriptive data of the composite use-frequency variables and their correlation with equivalent pleasantness variables are presented in Table 6. Except for the bitter vegetable and pungent foods variables, all correlations were strong (correlation coefficients 0.389–0.726) and significant (*p* < 0.001). The correlation between bitter vegetable liking and consumption was significant, but the correlation coefficient was only 0.238. Pungent food consumption was not correlated with liking. Cronbach’s alphas indicated a good internal consistency of the new use-frequency items except for bitter-and-astringent non-alcoholic beverages and sweet beverages.

The use-frequency components were also subjected to multivariate linear regression to reveal which factors predicted consumption other than the pleasantness score. The equivalent pleasantness score was the sole contributor to the model for every use-frequency component other than bitter vegetables, pungent foods, berries, fruits, and salty-and-savory foods. For these components, the regression models are presented in Table 7.

In addition to a higher pleasantness score, older age predicted an increased consumption of bitter vegetables. The contribution of bitter vegetable pleasantness score was only slightly higher than the contribution of age (standardized β coefficients of 0.280 and 0.249, respectively). The pleasantness score of pungent foods was not an important contributor to the consumption of pungent foods. Instead, male sex and low sensitivity to bitter taste were significant predictors for increased consumption of pungent foods (standardized β coefficients of −0.160 and −0.207, respectively). When the taste sensitivity score was applied in the model instead of the separate taste sensitivities, it had a significant contribution to the pungent foods consumption (F (df = 3, 144) = 4.00, *p* = 0.010, *R*^2^ = 0.076, β = −0.324); the less sensitive subjects consumed higher amounts of the pungent items.

The order of significance for factor contributions to berry consumption was pleasantness score (standardized β coefficient 0.574), BMI (−0.186), and age (0.127). The fruit pleasantness score had a higher contribution (standardized β coefficient 0.558) to the fruit consumption model than BMI (−0.193). The pleasantness score had the highest contribution (standardized β coefficient 0.664) to the salty-and-savory foods consumption model followed by sour sensitivity (0.173), umami sensitivity (−0.158), and sex (−0.137).

## 4. Discussion

In this study, the associations between taste sensitivity, food consumption behavior, and recalled pleasantness were investigated with 199 adult participants. To the authors’ knowledge, this is the first time that the perception of five taste modalities has been investigated in relation to food consumption and pleasantness. Thus far, research has been focused on the relationship between PROP and food-related behavior, whereas other tastants or taste qualities have gained only little attention. Additionally, large-scale studies with a varied group of subjects are scarce.

The consumption habits regarding some items were related to taste sensitivity. However, taste sensitivity was not related to the recalled pleasantness of the foods and beverages. As was expected, pleasantness was the main predictor of the use-frequency components, except for pungent foods. These results highlight the importance of studying the actual behavior toward food and not just liking.

### 4.1. Taste Sensitivity, Food Consumption, and Pleasantness

Bitter sensitivity was related to masking bitter tastes and pungent food consumption. Earlier studies on pungency or spicy foods behavior have focused on PROP taste. Higher sensitivity to PROP has been reported to predict a more intense pungency perception [12,23,30]. In a large scale study, the pungency of pure capsaicin correlated with PROP bitterness and taste intensities of other tastants, although the correlation coefficients were rather weak, at least for PROP (0.199) [12]. In the same study, PROP tasters perceived pungency in a food matrix more strongly than non-tasters. PROP bitterness perception was not related to the perception of oral pungency in another sample of Finnish subjects [31]. Intensity perception of oral pungency and liking of oral pungency or spicy foods have been shown to associate negatively when the subjects were grouped into pungency likers, medium-likers, and non-likers, although the correlation between intensity and liking was only −0.16 [31]. If bitter and pungent sensitivities correlated, bitter sensitivity could also associate negatively with pungent food liking. However, this theory was not supported in our study, as bitter sensitivity did not explain pungent food pleasantness. However, bitter sensitivity explained pungent food consumption, as bitter sensitive subjects reported eating pungent items less frequently.

Masking a bitter taste in food or modifying the taste of food by bitter sensitive subjects was shown in the habits of adding milk to coffee, and adding ketchup to a meal when eating it. Additionally, the habit of consuming coffee was less common among bitter sensitive subjects. However, the pleasantness or use-frequency of bitter non-alcoholic beverages, including coffee, was not related to bitter sensitivity. The relationship between coffee consumption and bitter sensitivity has been shown earlier with other kinds of study design. Lipchock et al. [17] showed, though with a small sample size, that daily coffee drinkers rated the intensity of pure caffeine higher than those who consumed coffee irregularly or not at all. Among Italian subjects, PROP sensitivity was not related to coffee liking, but interestingly, the PROP non-tasters added sugar to coffee more frequently than medium or supertasters although they perceived the coffee as milder than others did [32].

Some findings were surprising and challenging to explain. Sour sensitivity was related to the habits of adding sugar/honey to tea and soy sauce to a meal as well as to the consumption frequency of foods with salty and savory dominant tastes. Umami sensitivity was also related to the habit of adding sweetness to tea. Adding ketchup to a meal was also more common among the most sensitive to salt and sweetness. Ketchup is typically a strong-tasting sauce that contains spices, vinegar, sugar, and salt. The considerable amounts of sugar and salt in ketchup may explain why salty and sweet sensitivities were related to ketchup consumption. Concerning these results, there are no other published studies to compare so far. Thus, more large-scale studies are needed.

Umami sensitivity was related to umami-tasting foods: salty-and-savory foods, and weekly vegetable consumption. The result that lower umami sensitivity was related to more frequent consumption of umami-containing foods supports the idea that lower sensitivity to a taste demands higher concentrations of equivalent tastants to reach liking and increased consumption. This idea may depend on a food matrix or a taste as the relationship was opposite regarding ketchup and bitter, salty, or sweet sensitivity. In the case of vegetables, the most sensitive to umami consumed more portions of vegetables per week than the least sensitive. As umami is also part of vegetable taste profiles [33] and umami intensity is affected by the processing of vegetables [34], umami sensitive people might perceive a more intense umami taste from vegetables, making them more palatable.

The descriptor of overall taste sensitivity, the taste sensitivity score, was related to three items only: consumption of pungent foods, coffee, and adding ketchup to a meal. These findings indicate that studying taste modality-specific sensitivities rather than general indicators of taste sensitivity might give better insights into the relationship between taste perception and food-related behavior.

Based on earlier studies, other associations may have existed. First, in our results, bitter (caffeine) sensitivity was not related to either vegetable liking or consumption, a tendency that has earlier been explained with PROP sensitivity [21,23] and one which, in turn, has been shown to associate with caffeine perception [32,35]. Therefore, there could have been some association with bitter sensitivity and vegetable-related behavior in this study, too.

Second, taste sensitivity was not related to alcohol pleasantness or intake. An earlier study found that perceived NaCl and sour taste intensities correlated positively with yearly alcohol intake [18]. In multiple regression analysis, they were not significant predictors of intake, but PROP intensity perception predicted alcohol intake as lower sensitivity indicated higher intake [18]. It must be noted that in our study, the consumption of alcoholic beverages was low, which may contribute to the results.

Third, sweet sensitivity could have been related to sweet-and-fatty foods or sweet beverage/sweet item liking and/or consumption. Jayasinghe et al. [7] reported that among New Zealand women, a higher sweet sensitivity indicated lower consumption frequency of baking/sweets (e.g., chocolates, biscuits, cakes) which were reported to be consumed more often than once per day. Furthermore, total sweet food intake was lower (on average seven times per day) among sweet sensitive subjects, as well as liking of fruit drinks and fruit juices when liking of 16 sweet beverages was measured. On the contrary, Low et al. [8] found that sweet taste sensitivity was not related to intake of total sugars, added sugar, or sugar-sweetened foods. Additionally, sweetness sensitivity was not related to sweet food liking or consumption in other studies [15,36]. Furthermore, among young adults, sweet perception was not related to sweet food behaviors including intake of confectionery, fruits, or vegetables, or the importance of adding sugar to tea or coffee or avoiding sugar-sweetened or fizzy drinks [16].

Tepper [23] reviewed links between PROP tasting and food-related behavior. The links were not confirmed as there were discrepancies in results between studies. A vast range of methods can explain some discrepancies. Some studies show that the association between PROP tasting and food consumption behavior may depend on sex, age, fungiform papillae density, or personality trait [21,23]. It seems that PROP tasters can perceive more intensively or differentiate some other properties more easily than non-tasters, but this might not always translate into hedonics or consumption of foods [23]. However, earlier studies have found that PROP sensitivity might negatively affect behavior related to pungent, bitter, and creamy foods [23]. In a more recent study, a higher PROP bitterness perception was related to a lower liking and consumption of not only bitter but other vegetables among young adults [21]. Catanzaro et al. [19] found no significant association between PROP tasting and recalled liking of foods that have been reported to be related to PROP taster status. In their study, PROP intensity correlated statistically significantly with the liking of dark chocolate and chili peppers, but the correlation coefficients were only −0.155 and −0.144, respectively.

This study was cross-sectional; thus, no cause and effect relationship can be concluded. A study by Wise et al. [37] has indicated that reduced sugar consumption causes a more intense sweetness perception in a food matrix but does not affect pleasantness ratings. In the case of salt, salt perception did not change but preference for higher levels of salt increased with increasing salt intake [38]. In a more recent study, perception or pleasantness was not affected by the intake of salt [39]. Noel et al. [40] showed that repeated exposure to MSG in broth diminished umami intensity perception, as well as desire for and intake of savory foods at an ad libitum meal.

### 4.2. Background Factors, Food Consumption, and Pleasantness

Sex and age, as well as BMI in some cases, were related to pleasantness components, but they explained only a relatively small proportion of pleasantness in regression models. They were also related to many food consumption variables.

In our study, females liked pungent foods more but males consumed them more. Earlier, pungent food liking [41] and chili pepper consumption [30] were related to the male sex, and spicy food consumption has been shown to be independent of sex [42]. Törnwall et al. [31] also found that genetic factors could explain 18–58% of liking and perception of pungency and spicy foods. Additionally, some personality factors, such as food adventurousness, can explain spicy food consumption and liking [23,30,31]. We found no connection between pungent food pleasantness and consumption, although it would be logical that those who like pungency would consume it more than those who dislike it. Ludy and Mattes [42] found this logic with a small sample size, as regular spicy food users liked chili pungency and spicy foods more than non-users. They also found that many of the users had been already introduced to spicy foods in childhood, indicating the relevance of early and repeated exposure to food-related behavior. It should be noted that in our study, a limited number of pungent foods was included.

There were also other sex-related differences. Males liked bitter-and-astringent alcohol more and consumed salty-and-savory items more frequently, while females favored sweet-and-fatty foods. Earlier studies have also found that Finnish females liked sweet-and-fatty foods or sweet foods more than males did [41,43]. Valsta et al. [1] reported that women had consumed more VFB than men during recent years. We found only that females ate more portions of berries per week. In use-frequency components, sex did not have a significant role in explaining the consumption of VFB, but males had higher scores for bitter vegetable pleasantness.

As age increased, the pleasantness score increased for strong-tasting vegetables, berries, salty-and-savory foods, and bitter non-alcoholic beverages, whereas the younger subjects liked salty-and-fatty foods and sweet beverages more. Valsta et al. [1] showed that in Finland, the number of people consuming the recommended amount of vegetables decreases and the number of people consuming the recommended amount of fruits and berries increases by age. In accordance with Valsta et al. [1], we found the consumption of fruits (portions per week) and berries (use-frequency) to increase by age, but we also found an increase by age in the use-frequency (components) of bitter vegetables.

The older subjects seemed to like and consume more strong tastes as bitterness was not a barrier for liking, and those foods with a strong umami taste were considered pleasant. The younger subjects’ avoidance of bitterness was supported by the frequency with which they added milk to coffee. The oldest subjects who were also less sensitive to sour used to add soy sauce to a meal when eating it more frequently than more sensitive subjects. One explanation could be that the oldest subjects try to compensate for their weakened taste sensitivity by adding soy sauce to food. The oldest subjects also added sugar to berries more frequently than the younger participants despite their taste sensitivity, which might explain why older age was related to a higher liking and consumption of berries. Among Finnish consumers, increased berry liking has been linked to female sex and older age as well as some personality traits [44]. In this study population, the older subjects were less taste sensitive [29] which could explain the liking of strong and bitter-tasting foods and beverages. However, taste sensitivity was not a significant predictor in regression analysis. Another possible explanation might be becoming accustomed to strong tastes after repeated exposure with age. Then again, cultural and social aspects could explain why the younger participants liked more salty-and-fatty foods and sweet beverages. Coffee culture in Finland has evolved, and younger participants might be becoming used to consuming their coffee with milk, such as in cappuccinos or lattes.

BMI was related to the consumption frequency of berries (both weekly portions and use-frequency component), the pleasantness of bitter vegetables, and the pleasantness and consumption frequency of fruits; the lower the BMI, the higher the score for these variables. In contrast, those subjects with a higher BMI liked sweet beverages more. These results reflect the assumption that people with a lower BMI might have a healthier diet. However, BMI was not a significant predictor for salty-and-fatty or sweet-and-fatty food consumption or pleasantness. In a recent study, Low et al. [36] found no correlation between BMI and sweet food liking or consumption. In the study by Guido et al. [45], BMI was not related to the preference variables formed with factor analysis: vegetables, fruits, spicy, and milk products. Likewise, there were no differences between lean and obese subjects in the liking of foods with different predominant taste qualities. In an earlier study, the lean subjects liked salty/savory and sweet foods more than the obese subjects did [46].

Education level was related only to the habits of adding milk to coffee and sugar or honey to tea. Education could have been related more extensively to food consumption because education is one indicator of socioeconomic position, which can have an impact on dietary habits [47]. Valsta et al. [1] also reported differences in VFB consumption between education levels as the better-educated people consumed more of these than the lower educated ones. In this study, no relationship was found between education and the consumption of VFB. This might be due to the larger proportion of better-educated participants in this study.

### 4.3. Limitations

This study considered five taste modalities to explain food consumption and food pleasantness among Finnish adults. The subjects formed a large heterogeneous group of consumers and a wide variety of variables about food consumption behavior was collected. However, there are some limitations to acknowledge. The sample population was unbalanced for background factors. Additionally, the taste sensitivity groups were different in size as a result of the data-driven determination. Although the number of males and obese subjects were smaller than their reference groups, the numbers were higher than those in many earlier studies. A representative population sample was not our aim, but the characteristics of the sample population should be kept in mind when interpreting the results.

The questionnaires about food consumption behavior and pleasantness were not validated. Existing questionnaires, such as the French PrefQuest [48], are not valid globally, as food consumption is strongly connected to culture. Thus, such a questionnaire could not be applied to study food consumption behavior or pleasantness among Finnish people, and we had to develop a new one. The food items in this study were chosen based on the expectation that they would divide people’s opinions and elicit different taste sensations.

Concerning the taste sensitivity determination, only one prototypic compound per taste quality was used. With other compounds, the results might have been different. On the other hand, Mojet et al. [49] found no compound-specific differences within taste modalities between sexes or age groups. No references were used to guide the intensity evaluations, but thorough written and verbal instructions were given on how to use the scale. It is not guaranteed that individual ratings were the results of true intensity perception only and not results of scale-use bias. The sensitivity groups were formed via hierarchical clustering, and simultaneous analyses of individual’s evaluations made it possible to smooth out variation in sample rating.

## 5. Conclusions

Taste sensitivity was related to some food consumption behavior; not to recalled pleasantness but to use-frequency, and tendency to mask or modify tastes. Thus, the focus of research should be in studying actual behavior toward food and not just liking. All taste modality-specific sensitivities were related to some aspect of food consumption behavior. The taste sensitivity score – describing overall taste sensitivity – was related only to pungent food consumption, coffee drinking, and the habit of adding ketchup to food. These findings imply that it would be more informative to study the associations between taste sensitivity and food-related behavior with all taste modalities separately rather than with any general indicator of taste sensitivity. Sex, age, and BMI were related to several food consumption habits and pleasantness, but only a small proportion of food pleasantness was explained by them. Additionally, pleasantness was the main factor explaining consumption frequency. Clearly, factors other than taste sensitivity are also important for food liking and consumption. Culture and social dynamics may have a significant role in how an individual perceives and approaches foods and beverages. Fortunately, food choice and intake can be affected by encouraging healthier choices, and after several exposures people can learn to like, for example, vegetables, fruits, and berries, rather than the preference being determined by biology. There are substantial cultural differences in the ways and frequency of how foods and beverages are consumed. Thus, more studies are needed to fully understand the importance of taste perception in actual behavior toward food.

## Figures and Tables

**Table 1:** Taste samples.

Taste	Prototypic Tastant	Sample A (mM)	Sample B (mM)	Sample C (mM)	Sample D (mM)
Sour	Citric acid ^1^	3.33	1.87	1.05	0.57
Bitter	Caffeine ^1^	3.60	2.03	1.14	0.62
Sweet	Sucrose ^2^	58.4	32.9	18.5	10.5
Salty	Sodium chloride (NaCl) ^1^	34.2	19.2	10.8	5.99
Umami	L-glutamic acid, monosodium salt (MSG) ^1^	10.7	6.01	3.38	1.87

^1^ Produced by Sigma-Aldrich, St. Louis, USA; ^2^ Produced by Alfa Aesar GmbH&Co KG, Karlsruhe, Germany.

**Table 2:** Subjects’ characteristics (*N* = 199).

Variable	*n*	%	Data Missing (*n*)
Age	199		0
19–34 years	86	43.2	
35–49 years	56	28.1	
50–79 years	57	28.6	
Sex	199		0
Female	158	79.4	
Male	41	20.6	
BMI	192		7
<25.0	110	57.3	
25.0–29.9	49	24.6	
≥30.0	33	17.2	
Education ^1^	196		3
Low	73	37.2	
High	123	62.8	
Sour sensitivity	197		2
Least sensitive	49	24.9	
Semi-sensitive	101	51.3	
Most sensitive	47	23.9	
Bitter sensitivity	196		3
Least sensitive	35	17.9	
Semi-sensitive	83	42.3	
Most sensitive	78	39.8	
Sweet sensitivity	199		0
Least sensitive	80	40.2	
Semi-sensitive	79	39.7	
Most sensitive	40	20.1	
Salty sensitivity	198		1
Least sensitive	112	56.6	
Semi-sensitive	51	25.8	
Most sensitive	35	17.7	
Umami sensitivity	198		1
Least sensitive	29	14.6	
Semi-sensitive	132	66.7	
Most sensitive	37	18.7	

^1^ Low education included comprehensive school, high school, and lower vocational degree, whereas high education included a polytechnic degree or any university degree.

**Table 3:** Distribution of responses [*N* (%)] for habits of masking/modifying taste.

	Add Milk to Coffee	Add Sugar/Honey to Tea	Add Salt to Vegetable Cooking Water	Add Salt to a Meal When Eating It	Add Ketchup to a Meal When Eating It	Add Soy Sauce to a Meal When Eating It
always	83 (52.2)	29 (15.7)	37 (19.5)	6 (3.1)	0 (0.0)	0 (0.0)
often	15 (9.4)	29 (15.7)	47 (24.7)	24 (12.6)	8 (4.2)	6 (22.2)
occasionally	7 (4.4)	31 (16.8)	40 (21.1)	37 (19.4)	70 (36.5)	42 (22.2)
rarely	16 (10.1)	44 (23.8)	29 (15.3)	83 (43.5)	72 (37.5)	70 (37.0)
never	38 (23.9)	52 (28.1)	37 (19.5)	41 (21.5)	42 (21.9)	71 (37.6)
Total *N*	159	185	190	191	192	189

**Table 4:** Rotated variable loadings of the extracted pleasantness components (correlation coefficients). The bolded coefficient indicates the highest correlation of the item. For simplicity, only coefficients above 0.400 are shown. The labels of new variables are in italics and the mean [SD] of the original pleasantness ratings (1 = extremely unpleasant, 9 = extremely pleasant) in the parentheses.

	PC1	PC2	PC3
Vegetables and pungent items (*N* = 154)			
*Bitter vegetables (6.46 [2.12])*			
Red beet	**0.725**		
Swedish turnip	**0.714**		
Brussels sprout	**0.710**		
Carrot	**0.530**		
Radish	**0.509**	0.401	
*Strong-tasting vegetables (6.37 [2.46])*			
Onion		**0.765**	
Rucola		**0.684**	
Olive		**0.658**	
Celery		**0.652**	
*Pungent foods (5.92 [2.37])*			
Chili sauce			**0.928**
Chili			**0.842**
Wasabi			**0.715**
Mustard			**0.448**
Variance explained (%)	28.9	12.8	7.7
Berries and fruits (*N* = 186)			
*Berries (7.20 [1.97])*			
Lingonberry	**0.833**		
Red currant	**0.817**		
Black currant	**0.754**		
Sea buckthorn berry	**0.658**		
Bilberry	**0.499**		
*Fruits (6.73 [2.04])*			
Avocado		**0.806**	
Lemon		**0.764**	
Rhubarb		**0.638**	
Grapefruit	0.432	**0.475**	
Variance explained (%)	33.7	10.4	
Sweet, salty, and fatty (*N* = 177)			
*Salty-and-fatty foods (6.67 [1.99])*			
French fries	**0.824**		
Potato chips	**0.769**		
Mayonnaise	**0.709**		
*Sweet-and-fatty foods (7.67 [1.66])*			
Ice cream		**0.800**	
Sweet pastry		**0.723**	
Milk chocolate		**0.642**	
Candy		**0.547**	
*Salty-and-savory foods (6.68 [2.52])*			
Blue cheese			**0.821**
Dry-cured salmon			**0.744**
Soy sauce			**0.479**
Variance explained (%)	24.0	15.0	11.1
Beverages (N = 170)			
*Bitter-and-astringent alcohol (5.84 [2.52])*			
White wine	**0.757**		
Dry cider	**0.716**		
Red wine	**0.699**	0.409	
Long drink	**0.656**		0.438
Strong alcohol	**0.610**		
Beer	**0.548**	0.539	
*Bitter-and-astringent non-alcoholic (7.04 [2.22])*			
Carbonated water		**0.782**	
Tea		**0.668**	
Coffee		**0.555**	
*Sweet beverages (4.81 [2.41])*			
Soft drink			**0.828**
Light soft drink			**0.754**
Sweet cider			**0.538**
Variance explained (%)	29.9	17.6	10.9

PC refers to Principal Component. *N* refers to the number of subjects included in the analysis.

**Table 5:** The results of hierarchical multivariate linear regression, food pleasantness components as dependent variables: unstandardized β coefficients (95% confidence intervals) and model statistics.

Pleasantness Component ^1^	Sex ^2^	Age ^3^	BMI ^3^	Model Statistics
Bitter vegetables (*N* = 149)	−0.417 * (−0.802, −0.032)	0.149 (−0.050, 0.347)	−0.210 * (−0.418, −0.003)	F_df = 3, 145_ = 3.39, *p* = 0.020, *R*^2^ = 0.066
Strong-tasting vegetables (*N* = 149)	0.208 (−0.179, 0.595)	0.395 *** (0.201, 0.588)		F_df = 2, 146_ = 8.47, *p* < 0.001, *R*^2^ = 0.104
Pungent foods (*N* = 149)	0.596 ** (0.202, 0.990)	0.082 (−0.115, 0.279)		F_df = 2, 146_ = 4.67, *p* = 0.011, *R*^2^ = 0.060
Berries (*N* = 180)	−0.004 (−0.367, 0.359)	0.330 *** (0.157, 0.502)		F_df = 2, 177_ = 7.16, *p* = 0.001, *R*^2^ = 0.075
Fruits (*N* = 180)	−0.005 (−0.366, 0.356)	0.075 (−0.104, 0.253)	−0.254 * (−0.446, −0.062)	F_df = 3, 176_ = 2.28, *p* = 0.081, *R*^2^ = 0.037
Salty-and-fatty foods (*N* = 174)	−0.167 (−0.525, 0.191)	−0.277 ** (−0.456, −0.098)		F_df = 2, 171_ = 4.90, *p* = 0.009, *R*^2^ = 0.054
Sweet-and-fatty foods (*N* = 174)	0.450 * (0.086, 0.815)	−0.021 (−0.203, 0.161)		F_df = 2, 171_ = 3.05, *p* = 0.050, *R*^2^ = 0.034
Salty-and-savory foods (*N* = 174)	0.116 (−0.237, 0.470)	0.235 ** (0.059, 0.412)		F_df = 2, 171_ = 3.57, *p* = 0.030, *R*^2^ = 0.040
Bitter-and-astringent alcoholic (*N* = 165)	−0.604 ** (−0.961, −0.248)	0.078 (−0.100, 0.255)		F_df = 2, 162_ = 6.21, *p* = 0.003, *R*^2^ = 0.071
Bitter-and-astringent non-alcoholic (*N* = 165)	−0.266 (−0.620, 0.088)	0.227 *(0.051, 0.404)		F_df = 2, 162_ = 4.65, *p* = 0.011, *R*^2^ = 0.054
Sweet beverages (*N* = 165)	−0.187 (−0.546, 0.172)	−0.379 *** (−0.564, −0.194)	0.291 ** (0.094, 0.489)	F_df = 3, 161_ = 6.92, *p* < 0.001, *R*^2^ = 0.114

* *p* < 0.05, ** *p* < 0.01, *** *p* < 0.001; ^1^ *N* refers to the number of subjects included in the analysis; ^2^ Entered in the analysis as dummy variable: 0 = male, 1 = female.; ^3^ Entered in the analysis as a category variable with increasing age/BMI (see Table 1).

**Table 6:** The descriptives of use-frequency components and their correlation with equivalent pleasantness components.

	Descriptives				Correlation		
Use-Frequency Variable	*N*	Mean ^1^	SD	Cronbach’s α	*N*	Correlation with Pleasantness	Sig. (2-tailed) of Correlation
Bitter vegetables	191	2.86	0.60	0.653	154	0.238	0.003
Strong-tasting vegetables	190	3.33	0.83	0.619	153	0.389	<0.001
Pungent items	187	2.75	0.90	0.727	153	0.065	0.426
Berries	190	3.03	0.84	0.722	184	0.604	<0.001
Fruits	189	2.69	0.72	0.571	183	0.602	<0.001
Salty-and-fatty foods	191	2.64	0.74	0.668	176	0.572	<0.001
Sweet-and-fatty foods	190	3.49	0.69	0.505	175	0.493	<0.001
Salty-and-savory foods	190	2.76	0.81	0.444	175	0.672	<0.001
Bitter-and-astringent alcoholic	191	2.21	0.74	0.785	170	0.726	<0.001
Bitter-and-astringent non-alcoholic	192	4.29	1.07	0.232	170	0.704	<0.001
Sweet beverages	190	2.02	0.71	0.355	168	0.634	<0.001

^1^ Range from 1 (more seldom than a few times per year or never) to 6 (daily).

**Table 7:** The results of hierarchical multivariate linear regression, use-frequency components as dependent variables: unstandardized β coefficients (95% confidence intervals) and model statistics.

Use-Frequency Component ^1^	Sex ^2^	Age ^3^	BMI ^3^	Pleasantness	Bitter Sensitivity ^3^	Sour Sensitivity ^3^	Umami Sensitivity ^3^	Model Statistics
Bitter vegetables (*N* = 149)	0.176 (−0.059, 0.411)	0.190 ** (0.074, 0.306)		0.176 *** (0.079, 0.274)				F (3, 145) = 8.59, *p* < 0.001, *R*^2^ = 0.151
Pungent foods (*N* = 148)	−0.343 * (−0.683, −0.004)	0.067 (−0.111, 0.246)			−0.259 * (−0.466, −0.052)			F (3, 144) = 4.45, *p* = 0.005, *R*^2^ = 0.085
Berries (*N* = 178)	0.048 (−0.205, 0.301)	0.131 * (0.001, 0.262)	−0.207 ** (−0.342, −0.071)	0.489 *** (0.386, 0.592)				F (4, 173) = 28.3, *p* < 0.001, *R*^2^ = 0.396
Fruits (*N* = 177)	−0.075 (−0.292, 0.142)	0.042 (−0.065, 0.149)	−0.182 ** (−0.298, −0.065)	0.415 *** (0.326, 0.504)				F (4, 172) = 27.0, *p* < 0.001, *R*^2^ = 0.386
Salty-and-savory foods (*N* = 172)	−0.266 * (−0.484, −0.048)	0.036 (−0.079, 0.151)		0.535 *** (0.445, 0.625)		0.196 ** (0.059, 0.334)	−0.219 * (−0.392, −0.046)	F (5, 166) = 32.1, *p* < 0.001, *R*^2^ = 0.492

* *p* < 0.05, ** *p* < 0.01, *** *p* < 0.001; ^1^ *N* refers to the number of subjects included in the analysis; ^2^ Entered in the analysis as dummy variable: 0 = male, 1 = female; ^3^ Entered in the analysis as a category variable with increasing age/BMI/taste sensitivity (see Table 1).

## Supplementary Materials

The following are available online at https://www.mdpi.com/2304-8158/8/10/444/s1, Table S1: Recalled pleasantness of all food and beverage items that were included in the factor analysis.
